# Likely Additive Ergogenic Effects of Combined Preexercise Dietary Nitrate and Caffeine Ingestion in Trained Cyclists

**DOI:** 10.5402/2013/396581

**Published:** 2013-12-14

**Authors:** Michal K. Handzlik, Michael Gleeson

**Affiliations:** ^1^School of Life Sciences, The University of Nottingham Medical School, Queen's Medical Centre, Nottingham NG7 2UH, UK; ^2^School of Sport, Exercise and Health Sciences, Loughborough University, Loughborough, Leicestershire LE11 3TU, UK

## Abstract

*Aims*. To evaluate the possible additive effects of beetroot juice plus caffeine on exercise performance. *Methods*. In a randomized, double-blinded study design, fourteen healthy well-trained men aged 22 ± 3 years performed four trials on different occasions following preexercise ingestion of placebo (PLA), PLA plus 5 mg/kg caffeine (PLA+C), beetroot juice providing 8 mmol of nitrate (BR), and beetroot juice plus caffeine (BR+C). Participants cycled at 60% maximal oxygen uptake ($\dot{\text{V}}{\text{O}}_{2}$max) for 30 min followed by a time to exhaustion (TTE) trial at 80% $\dot{\text{V}}{\text{O}}_{2}$max. Saliva was collected before supplement ingestion, before exercise, and after the TTE trial for salivary nitrate, nitrite, and cortisol analysis. *Results*. In beetroot trials, saliva nitrate and nitrite increased >10-fold before exercise compared with preingestion (*P* ≤ 0.002). TTE in BR+C was 46% higher than in PLA (*P* = 0.096) and 18% and 27% nonsignificant TTE improvements were observed on BR+C compared with BR and PLA+C alone, respectively. Lower ratings of perceived exertion during TTE were found during 80% $\dot{\text{V}}{\text{O}}_{2}$max on BR+C compared with PLA and PLA+C (*P* < 0.05 for both). *Conclusions*. Acute preexercise beetroot juice coingestion with caffeine likely has additive effects on exercise performance compared with either beetroot or caffeine alone.

## 1. Introduction

Dietary supplement intake, in addition to training, appropriate nutrition, and advantageous gene polymorphisms, is recognized to confer exercise performance benefits [1–4]. Such supplements (e.g., caffeine, bicarbonate, creatine, and beta alanine) are widely consumed by athletes in many sports.

The role of caffeine as an ergogenic aid is well established. The study by Costill et al. [5] was the first to demonstrate improved time-to-exhaustion (TTE) exercise with acute preexercise ingestion of caffeine. Subsequent investigations reported that caffeine intake can improve cycling [6, 7], high intensity running [8, 9], and repeated sprint running performance [10]. A recent review on the effects of caffeine on exercise performance suggested that, at least in endurance sports, even small to moderate caffeine doses (2-3 mg/kg of body mass) are likely to confer performance benefits [11].

Caffeine induces numerous physiological effects. Although its stimulating effects on exercise performance in humans were initially thought to occur via increased fatty acid oxidation rates and muscle glycogen-sparing effect [5, 7], several studies reported conflicting findings [12, 13]. In addition to peripheral actions, caffeine was shown to reduce perception of effort during exercise, suggesting that some ergogenic effects may be partially accounted for by caffeine-mediated maintenance of central nervous system (CNS) drive [14]. Specifically, Meeusen et al. [15] implied that the performance-enhancing effects of caffeine are likely to be linked with the antagonism of adenosine receptor signalling in the brain.

Beetroot juice consumption has recently drawn attention of many endurance athletes as evidence accumulates indicating its performance-boosting effects. Several independent research groups have demonstrated in double-blind, placebo-controlled studies that acute as well as chronic intake of beetroot juice containing about 8 mmoles of nitrate (NO_3_^−^) can improve endurance and high-intensity intermittent performance [16–20]. Nitrate has been shown to be the key ergogenic ingredient of the beetroot juice. Following absorption, nitrate reappears, via enterosalivary circulation, in the mouth, where it is degraded to nitrite (NO_2_^−^) by facultative anaerobic bacteria on the surface of the tongue [21]. Further nitrite conversion to nitric oxide (NO) takes place in stomach [21]. Although the exact mechanisms by which increased beetroot juice-derived NO_2_^−^ and NO bioavailability enhance exercise performance remain to be fully elucidated, improved muscle mitochondrial function and/or reduced high-energy phosphate turnover leading to a decreased oxygen cost of exercise are likely to be involved [16, 19].

Considering that acute preexercise ingestion of caffeine and nitrate-rich beetroot juice has been found to enhance exercise performance via different mechanisms; their combined effects on performance could potentially be additive. In other words the preexercise ingestion of beetroot juice and caffeine could improve performance more than intake of either beetroot juice or caffeine alone. This is of obvious interest to athletes. There are relatively few studies on the effects of combining different dietary ergogenic aids, and to our knowledge, no study has investigated the potential combined ergogenic effects of nitrate and caffeine.

Therefore, the primary aim of this randomised, double-blind, placebo-controlled study was to evaluate potential additive effects of acute preexercise beetroot juice and caffeine supplementation on time to exhaustion during cycling at 80% $\dot{\text{V}}{\text{O}}_{2}$max in healthy, well-trained male participants. A secondary aim was determine if nitrate's effects on the oxygen cost of exercise are influenced by caffeine co-ingestion. We hypothesised that the combination of ingesting both beetroot juice and caffeine before exercise would have greater performance enhancing effects than beetroot juice or caffeine alone.

## 2. Methods

### 2.1. Participants

Fourteen well-trained male participants volunteered to participate in this randomized, double-blind, placebo-controlled design study. Their age, height, body mass, and $\dot{\text{V}}{\text{O}}_{2}$max were 22 ± 3 years, 177 ± 1 cm, 76 ± 7 kg, and 63 ± 10 mL/kg/min, respectively (values are expressed as mean ± SD). All participants engaged in endurance sports at least 3 times per week and all were familiar with cycling. All participants were fully informed of experimental procedures, associated risks, and a rationale of the study before they gave their written consent to participate in this study. Participants were included if they were nonsmokers, currently healthy, had been training for at least 2 h per week of moderate to vigorous physical activity, and had no known history of cardiac, hepatic, renal, pulmonary neurological, gastrointestinal, haematological, or psychiatric illness. The study had been earlier approved by the Ethics Committee of Loughborough University.

### 2.2. Preliminary Tests

All exercise tests were performed under controlled environmental conditions (22 ± 1°C, 50–60% relative humidity). Approximately one week before supplementation trials participants visited the laboratory to undertake screening, $\dot{\text{V}}{\text{O}}_{2}$max determination, and familiarization tests. Upon arrival, participants' weight was measured to the nearest 0.1 kg using a digital scale (Seca 770, Seca Ltd, Germany). Each subject performed a continuous incremental exercise test on one of the two electromagnetically braked cycle ergometers (Lode Excalibur Sport, Groningen, The Netherlands and Lode Corvial, Groningen, The Netherlands) to volitional exhaustion to determine $\dot{\text{V}}{\text{O}}_{2}$max. Subjects were randomly assigned to either one of the two cycle ergometers and the allocation was maintained during the supplementation trials. A fan was set approximately 2 m away from participants to provide cooling during exercise and supplementation trials. Participants pedalled at self-selected cadence between 60–120 rpm. Self-selected saddle and handlebar settings used were replicated for all subsequent supplementation trials.

Participants began cycling at 95 W, with increments of 35 W every 3 min until volitional fatigue despite strong verbal encouragement. Samples of expired gas were collected in Douglas bags during the third minute of each work rate increment, and heart rates were measured continuously using short-range telemetry (Polar, Kempele, Finland). A paramagnetic oxygen analyser (Servomex 1420B, Crowborough, UK) and an infrared carbon dioxide analyser (Servomex 1415B) were used to determine percentages of expired oxygen and carbon dioxide. These were used together with a dry gas meter (Harvard Apparatus, Edenbrige, UK) for a determination of rates of oxygen consumption ($\dot{\text{V}}{\text{O}}_{2}$) and carbon dioxide production ($\dot{\text{V}}$CO_2_). Maximal oxygen consumption was determined from the expired gas sample collected during the final minute of the exercise test when subjects indicated that they could only continue exercise for an additional minute. Work rates corresponding to 60% and 80% $\dot{\text{V}}{\text{O}}_{2}$max were interpolated from the $\dot{\text{V}}{\text{O}}_{2}$-work rate relationship. After 30 min recovery participants completed a 30 min familiarization trial at 60% $\dot{\text{V}}{\text{O}}_{2}$max, which followed the same format as the experimental trials. This was undertaken to ensure that the subjects were accustomed to the procedures used during the investigation and to minimise any potential learning or anxiety effects.

### 2.3. Experimental Exercise Tests

Following completion of the preliminary tests each participant visited the laboratory on four occasions interspersed by at least four days to complete 30 min exercise at 60% $\dot{\text{V}}{\text{O}}_{2}$max immediately followed by a time-to-exhaustion trial at 80% $\dot{\text{V}}{\text{O}}_{2}$max. The treatments on these trials were as follows: (1) placebo (PLA), (2) placebo + caffeine (PLA+C), (3) beetroot juice (BR), and (4) beetroot juice + caffeine (BR+C). The treatments were administered using a randomized double-blind, placebo-controlled study design. A BR+PLA trial was not included because that would have supplied only half of the required amount (8 mmoles) of nitrate.

On test days, participants arrived at the laboratory after an overnight fast in a rested state avoiding strenuous physical activity the day before at either 09:00 or 11:30. Their arrival time was maintained constant throughout the supplementation trial. After ~10 min rest they consumed a standardized breakfast containing 127 kcal (24 g of carbohydrate, 2.8 g of fat, and 1.6 g of protein) and a 70 mL concentrated beetroot juice (Beet-It, James White Drinks Ltd, Ashbocking, UK) containing 4 mmol NO_3_^−^ or placebo (nitrate-depleted Beet-It containing <0.1 mmol NO_3_^−^ from the same manufacturer). After 75 min participants consumed another 70 mL concentrated beetroot juice or placebo with or without caffeine (0.5 mg/kg body mass). Following another 75 min rest period subjects performed 30 min cycling at 60% $\dot{\text{V}}{\text{O}}_{2}$max followed by a time-to-exhaustion (defined as inability to maintain pedalling cadence) trial at 80% $\dot{\text{V}}{\text{O}}_{2}$max. During supplementation trials water was allowed ad libitum at all times. The timing and dosages of caffeine and nitrate were based on previous studies that have demonstrated ergogenic effects in endurance exercise with 5 mg/kg caffeine provided 1-2 hours before exercise [4–6, 9, 11, 13, 14] and 8 mmoles of dietary nitrate provided 2-3 hours before exercise [16–19, 22–24]. Expired gas samples data were collected for one minute during the tenth and twentieth minute of the initial 30 min exercise and during the seventh minute of the 80% $\dot{\text{V}}{\text{O}}_{2}$max time-to-exhaustion exercise along with participants' heart rate and rating of perceived exertion (RPE) collected every 5 min using the Borg 6–20 scale. Participants were unaware of the cycling duration during the trials and were given constant encouragement to cycle to exhaustion. Rates of whole-body fat and carbohydrate oxidation were calculated from measured gaseous exchange variables as previously described [25].

To help ensure that metabolic conditions were similar prior to each experimental trial, participants were asked to record dietary and fluid intake during the day before the first trial and replicate it during the day before the subsequent supplementation trials. Excessive caffeine (>5 cups of tea, coffee, and coke per day) and nitrate-rich foods (e.g. spinach, rocket, lettuce, and radish) were not permitted over the course of the study.

### 2.4. Saliva Collection and Analysis

Saliva samples, for salivary cortisol, nitrite, and nitrate determination, were collected before supplement ingestion, prior to exercise, during the last 5 min of the ride at 60% $\dot{\text{V}}{\text{O}}_{2}$max, and 10 min after completion of the time-to-exhaustion test. Water intake was not permitted 5 min prior to saliva collection at any time point to avoid salivary analyte dilution. Briefly, participants were seated and provided an unstimulated saliva sample by passive dribble into a 7 mL polypropylene collection tube (Sterilin, Caerphily, UK) over a course of 2–4 min with opened eyes, slightly bent forward with minimal orofacial movement. All samples were immediately centrifuged at 12 000 rpm for 2 min and the supernatant was collected and stored at −20°C for further analysis. The salivary cortisol concentrations were determined in duplicate using an ELISA kit (Salimetrics, State College, PA, USA). Duplicate salivary nitrate and nitrite were analysed using a colorimetric assay kit (Cayman Chemicals, USA). The intra-assay coefficient of variation for cortisol, nitrate, and nitrite was <3.6%.

### 2.5. Statistical Analysis

Data were tested for normal distribution using the Shapiro-Wilk test. RPE, physiological, and salivary cortisol variables were analysed using repeated measures ANOVA or Friedman ANOVA for normally and nonnormally distributed data, respectively. *Post hoc* Sidak or Wilcoxon signed rank test was used to detect significant differences between groups where these were apparent for normally and nonnormally distributed data, respectively. Statistical significance was accepted at *P* ≤ 0.05. Data in the text and tables are presented as mean and standard deviation (SD). All statistical analyses were carried out using SPSS 19.0 for Windows (SPSS, Chicago, IL, USA).

## 3. Results

### 3.1. Cardiorespiratory and Metabolic Parameters

Friedman's ANOVA revealed lower RPE during TTE exercise at 15 min of 80% $\dot{\text{V}}{\text{O}}_{2}$max on BR+C compared with PLA and PLA+C (*P* = 0.016 and *P* = 0.047, respectively) as shown in Table 1. There was a tendency for RPE to be reduced at 10 and 15 min of 80% $\dot{\text{V}}{\text{O}}_{2}$max on BR+C compared with PLA (*P* = 0.081) and BR (*P* = 0.084), respectively.

There was no interaction effect for HR during 30 min exercise at 60% $\dot{\text{V}}{\text{O}}_{2}$max and 80% $\dot{\text{V}}{\text{O}}_{2}$max and RPE during 30 min 60% $\dot{\text{V}}{\text{O}}_{2}$max exercise between the trials (data not shown; *P* > 0.05). No significant differences (*P* ≥ 0.104) between treatments were found in any other measured variable as shown in Tables 2 and 3.

### 3.2. Salivary Nitrite and Nitrate

**Figure 1:**
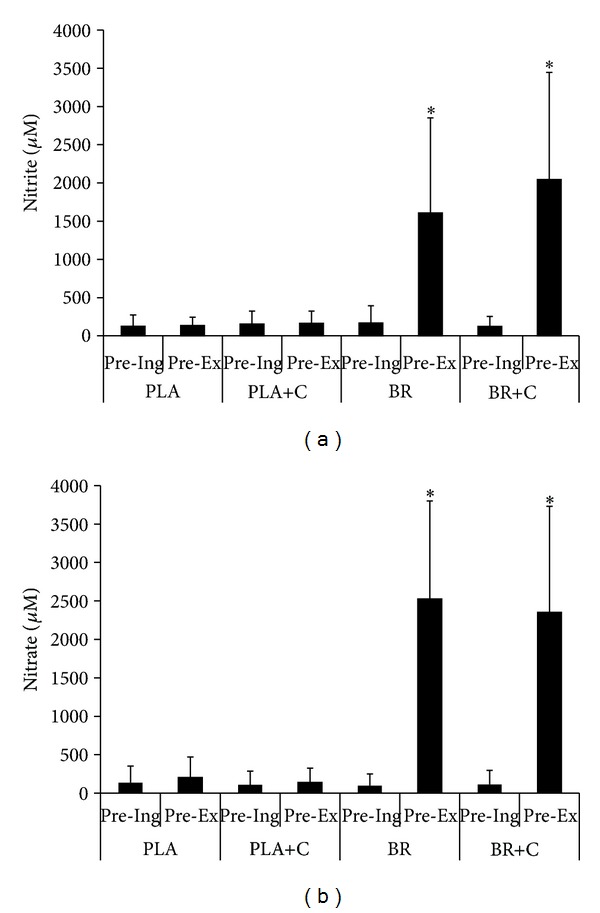
Salivary nitrite (a) and nitrate (b) before beetroot juice ingestion and preexercise on different supplementation trials. *Different from Pre-Ing in the same treatment; *P* ≤ 0.002. PLA: placebo; PLA+C: placebo plus caffeine; BR: beetroot juice; BR+C: beetroot juice plus caffeine; Pre-Ing: before ingestion; Pre-Ex: before exercise. Data are expressed as mean ± SD.

Resting preexercise salivary nitrite and nitrate concentrations were higher as shown by the paired *t*-test and Wilcoxon signed rank test after beetroot juice ingestion (*P* ≤ 0.002) than before (Figures 1(a) and 1(b)). On the contrary, no differences in salivary nitrate and nitrite were found between preingestion and preexercise samples in PLA and PLA+C (*P* > 0.05).

### 3.3. Time to Exhaustion at 80% $\dot{\text{V}}{\text{O}}_{\text{2}}$max

**Figure 2:**
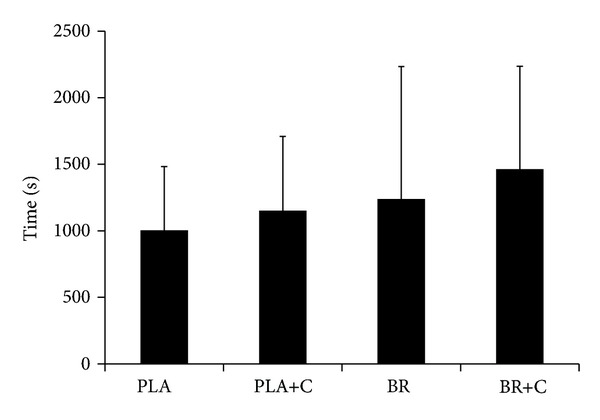
Time to exhaustion at 80% $\dot{\text{V}}{\text{O}}_{2}$max during four different trials. PLA: placebo; PLA+C: placebo plus caffeine; BR: beetroot; BR+C: beetroot plus caffeine. Data are expressed as mean ± SD.

**Figure 3:**
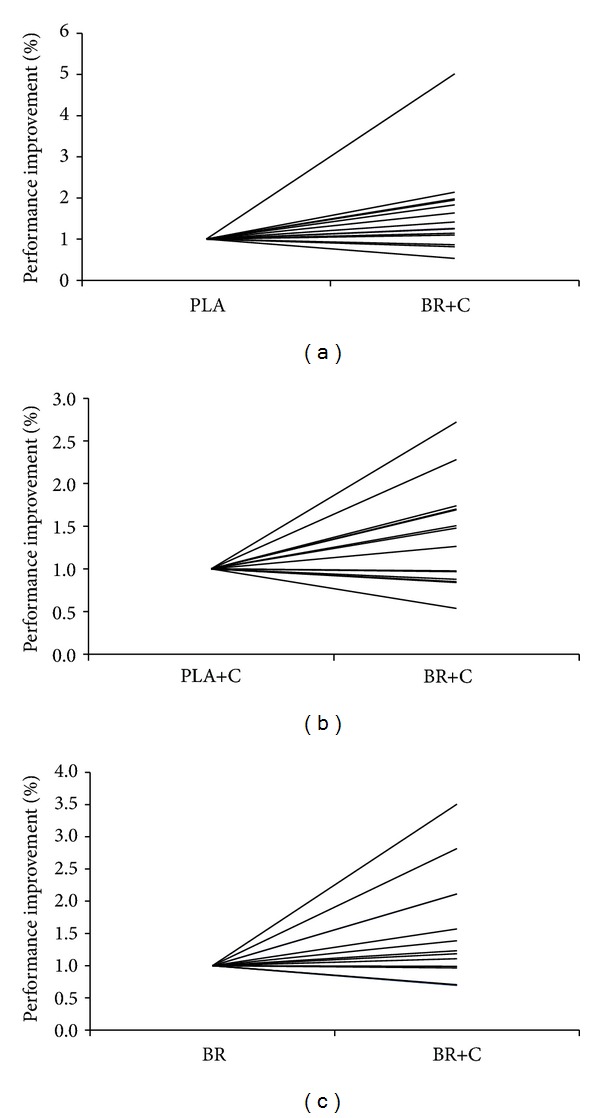
Individual responses on different supplementation trials. (a) PLA versus BR+C; (b) PLA+C versus BR+C; (c) BR versus BR+C. PLA: placebo; PLA+C: placebo plus caffeine; BR: beetroot juice; BR+C: beetroot plus caffeine.

No significant differences in time-to-exhaustion protocol (*F*_(3,39)_ = 2.269, *P* ≥ 0.096) between trials were found (Figure 2); however, exercise time in BR + C tended (*P* = 0.096) to be higher than in PLA (1463 ± 774 versus 1003 ± 480 s), corresponding to a 46% improvement. Similarly, 18% and 27% nonsignificant time-to-exhaustion improvements were observed on BR+C trial compared with BR and PLA+C alone, respectively. The individual performance data demonstrate that eleven, nine, and eight subjects of fourteen cycled longer on BR+C compared with PLA, BR, and PLA+C, respectively (Figure 3).

### 3.4. Salivary Cortisol

**Figure 4:**
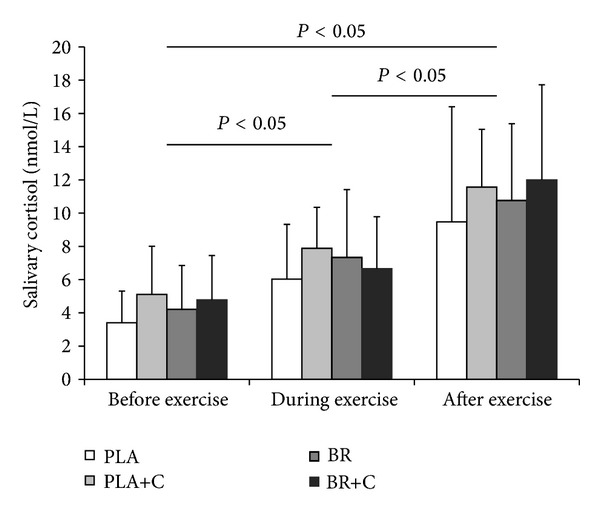
Salivary cortisol concentration before, during, and after exercise. PLA: placebo; PLA+C: placebo plus caffeine; BR: beetroot; BR+C: beetroot plus caffeine.

Salivary cortisol concentration is shown in Figure 4. Preexercise salivary concentration was not different between supplementation trials (*P* > 0.05). There was a main effect of time with salivary cortisol being higher during exercise compared to preexercise (*P* < 0.05). Likewise, salivary cortisol was found to be higher postexercise compared to preexercise and during exercise (*P* < 0.05) with no differences between supplementation trials (*P* > 0.05).

## 4. Discussion

The main purpose of the present study was to evaluate potential additive effects of beetroot juice and caffeine co-ingestion on TTE exercise performance. The other main goal of the experiment was to test the hypothesis if dietary nitrate reducing effects on oxygen uptake are influenced by caffeine ingestion. The major finding of this study was an 18% and 27% nonsignificant improvement in TTE at 80% $\dot{\text{V}}{\text{O}}_{2}$max on BR+C compared to BR and PLA+C, respectively. This finding supports our hypothesis implying potential positive and additive effects of BR+C on exercise capacity in well-trained male athletes. To our knowledge, this is the first study demonstrating potential beneficial effects of combined acute preexercise intake of beetroot juice and caffeine on TTE performance.

Our findings show that acute preexercise co-ingestion of beetroot juice and caffeine results in lower RPE, providing a potential mechanism, by which BR+C may improve exercise performance. Reduced RPE in BR+C compared to PLA and PLA+C suggests that beetroot and caffeine co-ingestion postponed the development of exercise intolerance and/or reduced perception of effort during exercise. As this finding was not observed for the PLA+C treatment, it is plausible that the reduction in RPE represents a BR+C-specific effect. The physiological importance of this finding is supported by the Doherty and Smith meta-analysis [14] indicating that caffeine-mediated improved exercise capacity can be largely explained by reduced RPE.

Preexercise caffeine intake alone has been suggested to activate neuroendocrine responses (e.g., noradrenaline secretion) to promote lipolysis and a glycogen-sparing effect [7, 10]. Results from studies using femoral arterial-venous catheters and muscle biopsy samples before and after exercise have called this caffeine-mediated glycogen-sparing effect into question [26, 27]. The findings of our study support this view. We did not observe any difference in salivary cortisol between trials, nor did we find any difference in whole-body carbohydrate or fat oxidation rates. These findings imply that acute preexercise caffeine intake alone or in combination with beetroot juice does not affect neuroendocrine responses and whole-body metabolic substrate utilisation during exercise in humans. Alternatively, a recent study indicated that caffeine may improve interstitial potassium handling during exercise, which is suggested to play a crucial role in fatigue development [10, 28]. Therefore, it can be argued that caffeine may have also acted via another peripheral mechanism that we did not measure.

The findings of the present study further support the ergogenic effects of beetroot juice intake on exercise performance in humans. For instance, in a randomized, cross-over design study, acute consumption of beetroot juice resulted in improved 4 km and 16 km time trial performance in club-level competitive cyclists [19]. Others have found that acute and chronic beetroot juice intake improves high-intensity TTE performance, while reducing oxygen uptake [22, 23]. Collectively, these findings suggest that both acute and chronic beetroot juice intake improve capacity to perform exercise in humans.

Beetroot juice-derived nitrate, nitrite, and NO are thought to act via multiple mechanisms. Dietary nitrate supplementation was shown to reduce the oxygen cost of exercise during both low- and moderate-intensity exercise in humans [16]. Using ^31^P magnetic resonance spectroscopy (MRS) chronic dietary nitrate supplementation attenuated the reduction in high-energy phosphate concentration (phosphocreatine) and reduced ATP turnover in humans during low- and high-intensity knee-extensor exercise, suggesting that the lower oxygen cost of exercise may be, at least partially, explained by a reduced ATP cost of muscle contraction [22]. In another study, Larsen et al. [24] investigated effects of chronic nitrate supplementation on human muscle mitochondrial function. Three days of sodium nitrate intake resulted in increased maximal ATP production by isolated mitochondria and reduced mitochondrial oxygen affinity [24]. Interestingly, during submaximal exercise, the nitrate supplementation treatments displayed lower oxygen consumption and higher RER suggesting more efficient energy utilisation. In mice, 7 days of sodium nitrate supplementation was shown to increase intact muscle fibre force production via greater intramuscular calcium concentration, suggesting another potential mechanism by which muscle performance may be enhanced [29]. Taken together, these findings suggest that increased nitrate bioavailability is involved in the regulation of intramuscular calcium handling, high-energy phosphate utilization, and oxidative metabolism.

More recently, however, the ergogenic effects of acute preexercise increased nitrate bioavailability have been questioned. Peacock et al. [30] reported that acute preexercise potassium nitrate consumption resulted in no improvement in 5 km time-trial performance in elite cross-country skiers. Further, Cermak et al. [31] found that consumption of a single dose of concentrated beetroot juice did not affect 1 h time-trial cycling exercise performance. In a similar fashion, a 3-day period of sodium nitrate supplementation was found to cause no improvement in distance covered or mean power output over a 40 min time-trial [32]. Although these studies imply that acute and/or chronic beetroot juice intake may not confer an exercise performance benefit, a recent review by Hoon et al. [33] indicates that, even if not reaching statistical significance, a small positive effect on time trial and graded exercise performance may be meaningful in an elite sport context.

The other main finding of the present study was that the oxygen cost of exercise at 60% and 80% $\dot{\text{V}}{\text{O}}_{2}$max on BR+C was unaffected by caffeine compared with beetroot juice alone, as might have been assumed if more free fatty acids were being oxidized following caffeine consumption (i.e., oxidation of free fatty acids requires ~10% more oxygen than carbohydrate to produce the same amount of energy). This finding demonstrates that caffeine alone or in combination with beetroot juice does not affect muscle substrate utilisation and oxygen consumption during exercise. Several potential factors may partially explain these discrepancies, including the exercise protocols used, participants' fitness levels, and the form of nitrate provided.

Although the overall magnitude of exercise improvement on BR+C may suggest physiological significance, no statistical difference between treatments was detected. This discrepancy may be, in part, explained by the study design, and thus statistical methods used to test for differences, and high interindividual variability in the TTE protocol. The individual performance data demonstrated that eleven out of fourteen and nine out of fourteen participants improved their exercise capacities on BR+C versus PLA and BR, respectively, suggesting a consistent general trend for performance enhancement on BR+C, yet a range of 2613 sec was observed between the longest and the shortest BR+C trial. This demonstrates that despite a general tendency for performance improvement, highly variable responsiveness exists between individual participants. This high interindividual variability may be, at least, partially explained by TTE protocol, in which participants were shown to display high test-retest variability. For instance, up to 27% versus <4% day-to-day variability has been reported for TTE and time-trial protocols, respectively, suggesting that further research is warranted to fully clarify effects of combined beetroot and caffeine intake on human exercise performance [24].

## 5. Conclusions

In summary, acute preexercise beetroot juice co-ingestion with caffeine augmented time-to-exhaustion at 80% $\dot{\text{V}}{\text{O}}_{2}$max performance by 18% and 27% over beetroot and caffeine alone, respectively. Moreover, 46% enhancement in exercise capacity was observed in BR+C compared with placebo, likely attributable to lower RPE in BR+C trial. There appears to be a strong likelihood that the ergogenic effects of dietary nitrate and caffeine are additive. Therefore, endurance athletes may benefit from this preexercise acute nutritional strategy prior to participation in competitive endurance events.

## Figures and Tables

**Table 1:** Ratings of perceived exertion at 80% $\dot{\text{V}}{\text{O}}_{\text{2}}$max.

	5 min	10 min	15 min	20 min	25 min	Exhaustion
PLA	16 ± 2	18 ± 2	19 ± 2	18 ± 2	18 ± 1	19 ± 1
PLA+C	16 ± 2	17 ± 1	18 ± 1	18 ± 1	18 ± 2	19 ± 1
BR	17 ± 2	17 ± 2	19 ± 1	17 ± 1	18 ± 1	19 ± 1
BR+C	16 ± 1	17 ± 2	17 ± 1*	18 ± 1	18 ± 1	19 ± 1

Data are expressed as mean ± SD. *Different from PLA and PLA+C; *P* ≤ 0.047. PLA: placebo; PLA+C: placebo plus caffeine; BR: beetroot; BR+C: beetroot plus caffeine.

**Table 2:** Cardiorespiratory and metabolic parameters during 60% $\dot{\text{V}}{\text{O}}_{\text{2}}$max exercise.

	PLA	PLA+C	BR	BR+C
HR (bpm)	152 ± 11	155 ± 10	151 ± 12	152 ± 13
${\dot{\text{V}}\text{O}}_{\text{2}}$ (L/min)	2.8 ± 0.4	2.8 ± 0.3	2.8 ± 0.4	2.9 ± 0.4
${\dot{\text{V}}\text{CO}}_{\text{2}}$ (L/min)	2.5 ± 0.3	2.6 ± 0.4	2.6 ± 0.4	2.6 ± 0.3
RPE	13.6 ± 1.9	13.1 ± 2.1	133 ± 1.7	12.9 ± 1.9
Carbohydrate oxidation (g/min)	2.25 ± 0.69	2.33 ± 0.83	2.56 ± 0.61	2.35 ± 0.81
Fat oxidation (g/min)	0.48 ± 0.33	0.46 ± 0.31	0.35 ± 0.20	0.48 ± 0.35
RER	0.90 ± 0.06	0.90 ± 0.06	0.93 ± 0.04	0.90 ± 0.06

Data are expressed as mean ± SD. HR: mean heart rate; ${\dot{\text{V}}\text{O}}_{\text{2}}$: mean oxygen uptake; ${\dot{\text{V}}\text{CO}}_{\text{2}}$: mean carbon dioxide production; RPE: mean ratings of perceived exertion; RER: respiratory exchange ratio.

PLA: placebo; PLA+C: placebo plus caffeine; BR: beetroot; BR+C: beetroot plus caffeine.

**Table 3:** Cardiorespiratory and metabolic parameters during 80% $\dot{\text{V}}{\text{O}}_{\text{2}}$max exercise.

	PLA	PLA+C	BR	BR+C
HR (bpm)	180 ± 7	168 ± 49	178 ± 9	181 ± 7
$\dot{\text{V}}{\text{O}}_{\text{2}}$ (L/min)	4.0 ± 0.4	4.0 ± 0.4	3.8 ± 0.5	4.0 ± 0.4
$\dot{\text{V}}\text{C}{\text{O}}_{\text{2}}$ (L/min)	3.8 ± 0.3	3.8 ± 0.5	3.8 ± 0.5	3.8 ± 0.4
Carbohydrate oxidation (g/min)	4.37 ± 1.18	4.21 ± 1.31	4.60 ± 1.06	4.24 ± 1.36
Fat oxidation (g/min)	0.23 ± 0.54	0.29 ± 0.45	0.06 ± 0.33	0.27 ± 0.56
RER	0.97 ± 0.08	0.95 ± 0.07	0.99 ± 0.05	0.96 ± 0.08

Data are expressed as mean ± SD. HR: mean heart rate; $\dot{\text{V}}{\text{O}}_{\text{2}}$: mean oxygen uptake; $\dot{\text{V}}\text{C}{\text{O}}_{\text{2}}$: mean carbon dioxide production; RER: respiratory exchange ratio. PLA: placebo; PLA+C: placebo plus caffeine; BR: beetroot; BR+C: beetroot plus caffeine.
